# Middle Interhemispheric Variant of Holoprosencephaly With Septo-Optic Dysplasia: A Rare Association

**DOI:** 10.7759/cureus.113295

**Published:** 2026-07-24

**Authors:** Jeremy R Luce, Johnathan Tran, Chetan Shah

**Affiliations:** 1 Pediatric Radiology, Lake Erie College of Osteopathic Medicine, Bradenton, USA; 2 Pediatric Radiology, Nemours Children's Health System, Jacksonville, USA

**Keywords:** brain malformation, cleft lip & palate, corpus callosum hypoplasia, diabetes insipidus, holoprosencephaly, middle interhemispheric variant, neural tube defect, optic nerve hypoplasia, septo-optic dysplasia, syntelencephaly

## Abstract

Middle interhemispheric variant (MIH) of holoprosencephaly (HPE), also known as syntelencephaly, is a rare subtype of HPE characterized by abnormal midline connection of the posterior parts of the frontal lobes and the anterior parts of the parietal lobes with variable corpus callosum abnormalities. We report the case of a five-year-old girl with syntelencephaly presenting with a cleft lip and palate, developmental delay, cerebral palsy, and intermittent diabetes insipidus. Brain magnetic resonance imaging demonstrated the characteristic features of both syntelencephaly and septo-optic dysplasia, including midline fusion of the frontal and parietal lobes, partial agenesis of the corpus callosum with hypoplastic genu and splenium, absence of the septum pellucidum, and bilateral optic nerve hypoplasia. Additional radiologic findings included bilateral subependymal gray matter heterotopia, colpocephaly, and an azygos anterior cerebral artery.

A literature search was conducted on PubMed and Google Scholar, with combinations of "middle interhemispheric variant" or "syntelencephaly" and "septo-optic dysplasia," "pituitary gland dysfunction," "endocrine dysfunction," or "optic nerve hypoplasia." To the best of our knowledge, this represents the first published case of MIH-variant HPE associated with all three criteria for septo-optic dysplasia, expanding the known phenotypic spectrum of these rare malformations. The co-occurrence of syntelencephaly and septo-optic dysplasia in this patient may reflect a shared disruption of midline forebrain development during the fourth to eighth weeks of gestation, when interhemispheric cleavage and hypothalamic-pituitary-optic development overlap temporally and depend on interconnected signaling pathways, including SHH, ZIC2, and the SOX family of transcription factors. Recognition of this association may prompt clinicians to evaluate patients with MIH variant HPE for features of septo-optic dysplasia, including optic nerve abnormalities and hypothalamic-pituitary dysfunction, and highlights the need for interdisciplinary clinical management and long-term surveillance in these patients.

## Introduction

Holoprosencephaly (HPE) is a congenital developmental disorder in which the forebrain fails to form normally during early embryogenesis, resulting in a spectrum of brain and craniofacial abnormalities [1,2]. It is the most common structural anomaly of the developing forebrain and is characterized by incomplete midline cleavage of the prosencephalon, meaning the primitive forebrain fails to divide completely into the right and left cerebral hemispheres [1,2]. The severity of this abnormal separation varies, ranging from partial to complete fusion of the cerebral hemispheres and other midline structures such as the corpus callosum, basal ganglia, and hypothalamus. Because the forebrain and midface both arise from the prechordal mesoderm, many patients with HPE also exhibit craniofacial abnormalities, including cleft lip and palate, microcephaly, microphthalmia, and cyclopia [2,3]. The development of HPE is multifactorial, with both genetic and environmental contributors, including maternal diabetes mellitus, in utero exposure to toxins, medications, infections, aneuploidy, and other genetic abnormalities [2,3].

HPE is classically divided into four subtypes based on the degree of nonseparation of the prosencephalon: alobar, semilobar, lobar, and the middle interhemispheric (MIH) variant, also known as syntelencephaly [1]. Originally described by Barkovich and Quint in 1993, syntelencephaly is a rare subtype of HPE characterized by an abnormal midline connection between the posterior parts of the frontal lobes and anterior parts of the parietal lobes, often accompanied by absence of the body of the corpus callosum [4]. Additional common findings include fusion of the thalami and caudate nuclei, gray matter heterotopias, cortical dysplasia, and an azygos anterior cerebral artery [1,4,5]. It is postulated to result from diminished migration of the mesenchyme to the midportion of the developing telencephalon due to ineffective mesenchymal production by the prechordal plate [4]. Recognition of HPE and its subtypes is clinically important because of the potential for significant neurologic, endocrine, visual, feeding, and developmental complications. These manifestations contribute substantially to morbidity and, in more severe forms of HPE, mortality, necessitating multidisciplinary evaluation, individualized management, and long-term follow-up.

Septo-optic dysplasia is a related congenital disorder characterized by abnormalities of midline brain development, underdevelopment of one or both optic nerves that can result in visual impairment or blindness, and pituitary dysfunction leading to endocrine abnormalities such as growth hormone deficiency, hypothyroidism, adrenal insufficiency, or diabetes insipidus [6]. The diagnosis is established when at least two of these three features are present [6]. Because visual and endocrine abnormalities may not be apparent at birth and can evolve over time, early recognition is essential to facilitate multidisciplinary management, hormone replacement when indicated, visual rehabilitation, and long-term surveillance to reduce morbidity.

## Case presentation

A five-year-old girl presented to our institution for preprocedural evaluation prior to direct laryngoscopy and bronchoscopy. She had been prenatally diagnosed with HPE based on fetal ultrasound findings of fusion of hemispheres across the midline. Brain magnetic resonance imaging (MRI) without intravenous contrast was obtained following physiologic myelination to further characterize the cerebral malformation.

She was born prematurely at 32 6/7 weeks' gestation with a birth length of 40.6 cm, weight of 1.56 kg, and head circumference of 29 cm. She was the product of her mother's sixth pregnancy, which was complicated by preeclampsia, intrauterine growth restriction, anemia, thrombocytopenia, and premature labor. There was no reported history of gestational diabetes, in utero exposure to infections, tobacco, or alcohol; however, the mother reported using unspecified anxiety medication during the first few weeks of pregnancy. There was no known family history of brain malformations, craniofacial abnormalities, or genetic disorders. One sibling was also born prematurely and had developmental delay, cerebral palsy, and a history of Wilms tumor of unknown etiology. At birth, the patient was noted to have a cleft lip and palate, a patent foramen ovale, and respiratory distress.

Over the ensuing years, she developed cerebral palsy with spasticity involving the upper and lower extremities, gastrostomy tube-dependent dysphagia, global developmental delay, laryngotracheomalacia, strabismus, amblyopia, recurrent respiratory infections, and intermittent diabetes insipidus treated as needed with desmopressin. At one year of age, ophthalmologic evaluation demonstrated decompensated V-pattern intermittent exotropia associated with inferior oblique overaction that was unresponsive to conservative management. She subsequently underwent bilateral lateral rectus recession (7.0 mm) and bilateral inferior oblique recession (10 mm). At the time of presentation, she weighed 16.2 kg (10th percentile) and measured 106.7 cm in height (18th percentile) [7].

Endocrine evaluation demonstrated elevated insulin-like growth factor-1 levels with normal growth hormone and insulin-like growth factor-binding protein-3 levels. Adrenocorticotropic hormone and cortisol levels were within normal limits. Previous genetic evaluation included chromosomal microarray analysis demonstrating a 133-kb gain at Xp22.33 encompassing the SHOX gene; because there was insufficient evidence supporting SHOX triplosensitivity, this finding was considered benign. Whole-exome sequencing identified a variant of uncertain significance consisting of an amplification of at least 51 kb within 15q15.3.

**Figure 1:**
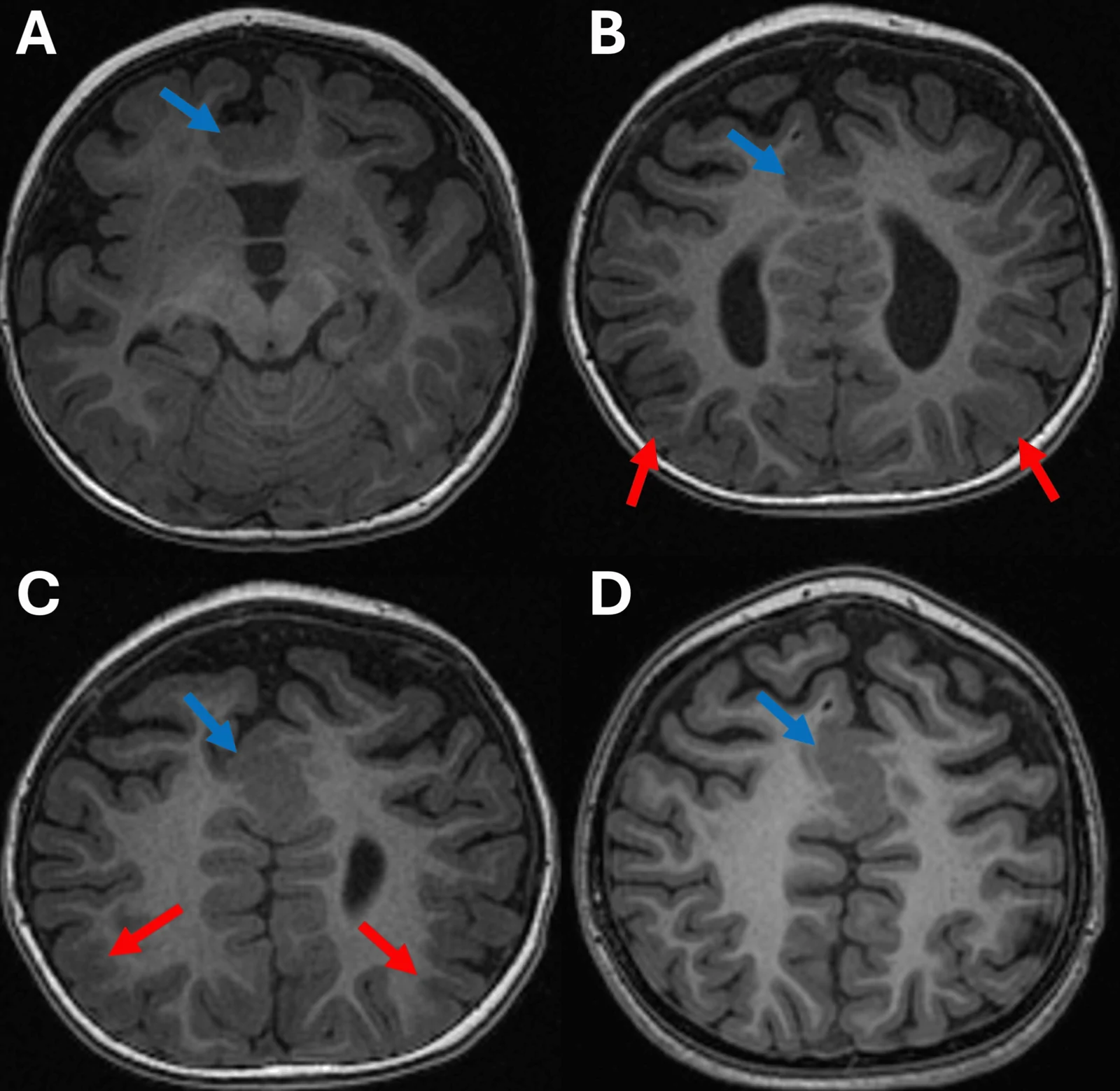
MRI images demonstrating midline fusion of the posterior parts of the frontal lobes and polymicrogyria Axial T1-weighted MRI images without intravenous contrast (A, D) show polymicrogyria (blue arrows) at abnormal fusion of the posterior parts of the frontal lobes. Axial T1-weighted MRI images without intravenous contrast (B, C) show polymicrogyria (blue arrows) at abnormal fusion of the posterior parts of the frontal lobes as well as abnormal sulcations and polymicrogyria bilaterally (red arrows).

**Figure 2:**
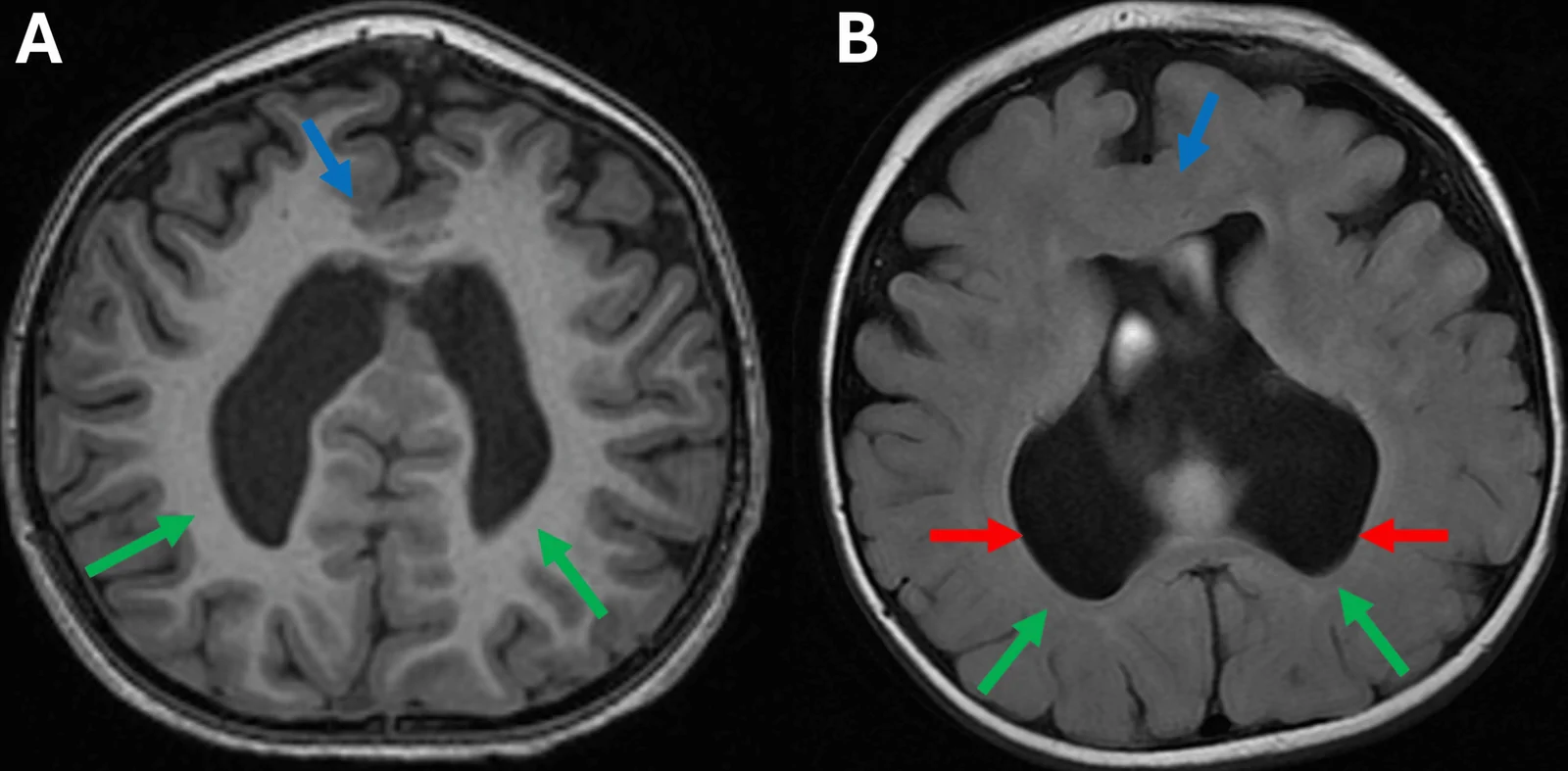
MRI images demonstrating midline fusion of the posterior parts of the frontal lobes, midline fusion of the anterior parts of the parietal lobes, colpocephaly, and thin periatrial white matter Axial T1-weighted MRI image without intravenous contrast (A) shows abnormal fusion of the frontal lobes (blue arrow) and thin periatrial white matter (green arrows). Axial fluid attenuation inversion recovery (FLAIR) weighted image (B) shows abnormal fusion of the frontal lobes (blue arrow), colpocephaly (red arrows), and thin periatrial white matter (green arrows).

**Figure 3:**
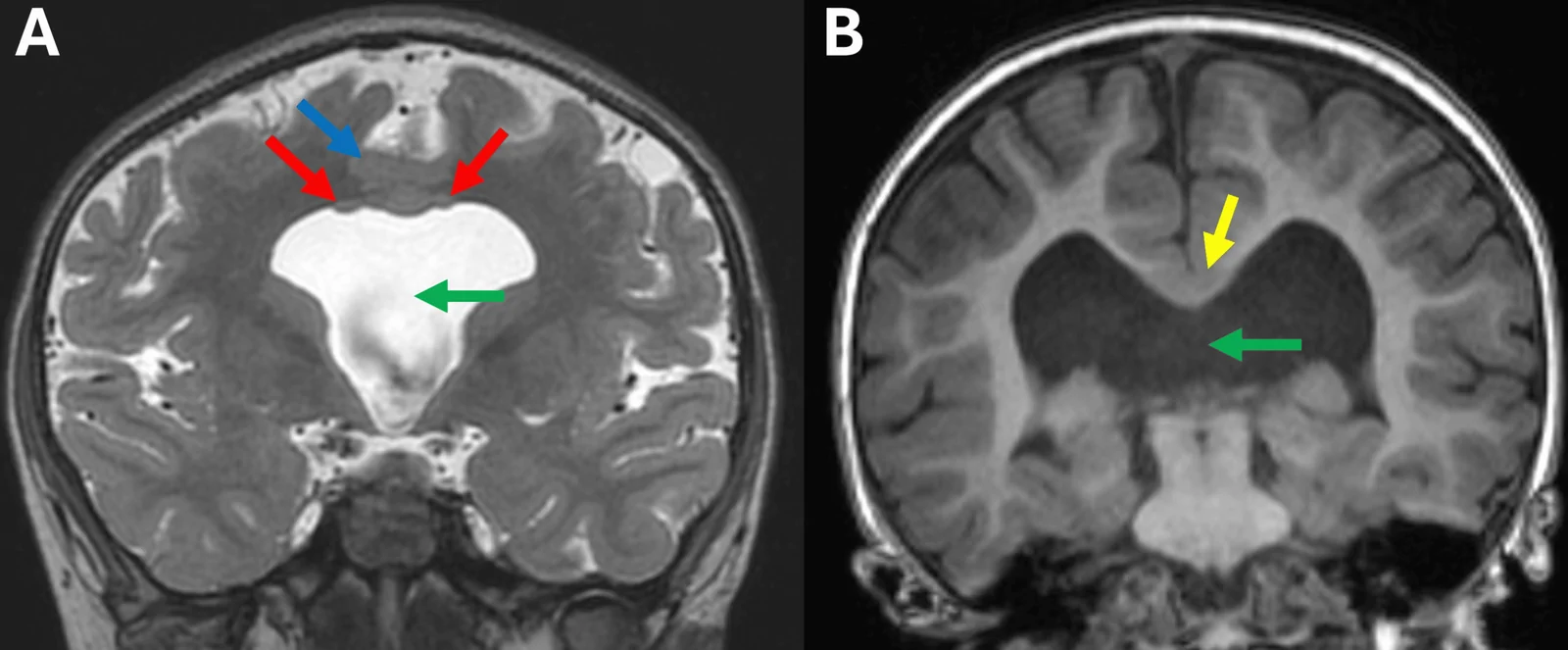
MRI images demonstrating midline fusion of the posterior parts of the frontal lobes, midline fusion of the anterior parts of the parietal lobes, subependymal gray matter heterotopia, and absence of the septum pellucidum Coronal T2-weighted MRI image (A) shows abnormal fusion of the frontal lobes (blue arrow), subependymal grey matter heterotopia (red arrows), and absence of the septum pellucidum (green arrow). Coronal T1-weighted MRI image without intravenous contrast (B) shows abnormal fusion of the parietal lobes (yellow arrow) and absence of the septum pellucidum (green arrow).

**Figure 4:**
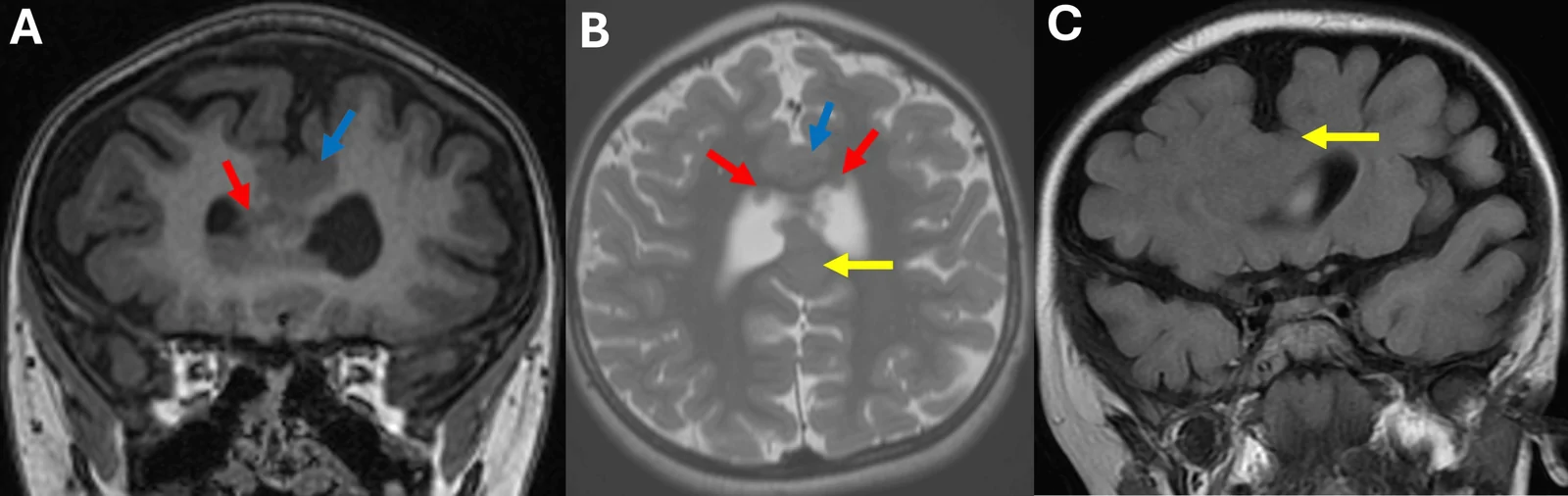
MRI images demonstrating midline fusion of the posterior parts of the frontal lobes, midline fusion of the anterior parts of the parietal lobes, and subependymal gray matter heterotopia. Coronal T1-weighted MRI image of the brain without intravenous contrast (A) shows abnormal fusion of the frontal lobes (blue arrow) and subependymal grey matter heterotopia (red arrow). Axial T2-weighted MRI image (B) shows abnormal fusion of the frontal lobes (blue arrow) and parietal lobes (yellow arrow) as well as subependymal grey matter heterotopia (red arrows). Coronal fluid attenuation inversion recovery (FLAIR) MRI image (C) shows abnormal fusion of the parietal lobes (yellow arrow).

**Figure 5:**
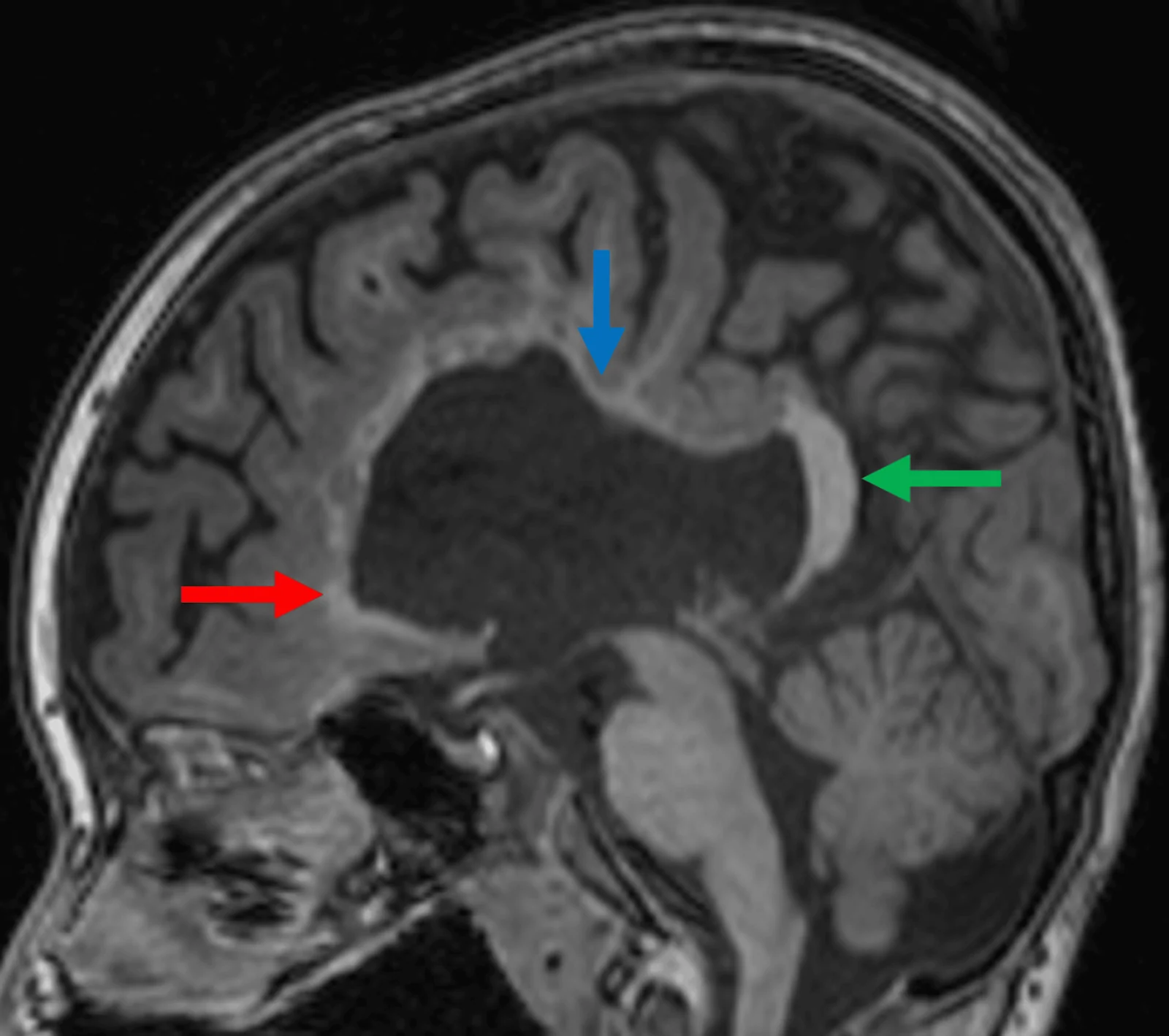
MRI image demonstrating absence of the body of the corpus callosum and hypoplasia of the genu and splenium of the corpus callosum Sagittal T1-weighted MRI image without intravenous contrast shows hypoplastic genu of the corpus callosum (red arrow), absent body of the corpus callosum (blue arrow), and hypoplastic splenium of the corpus callosum (green arrow).

**Figure 6:**
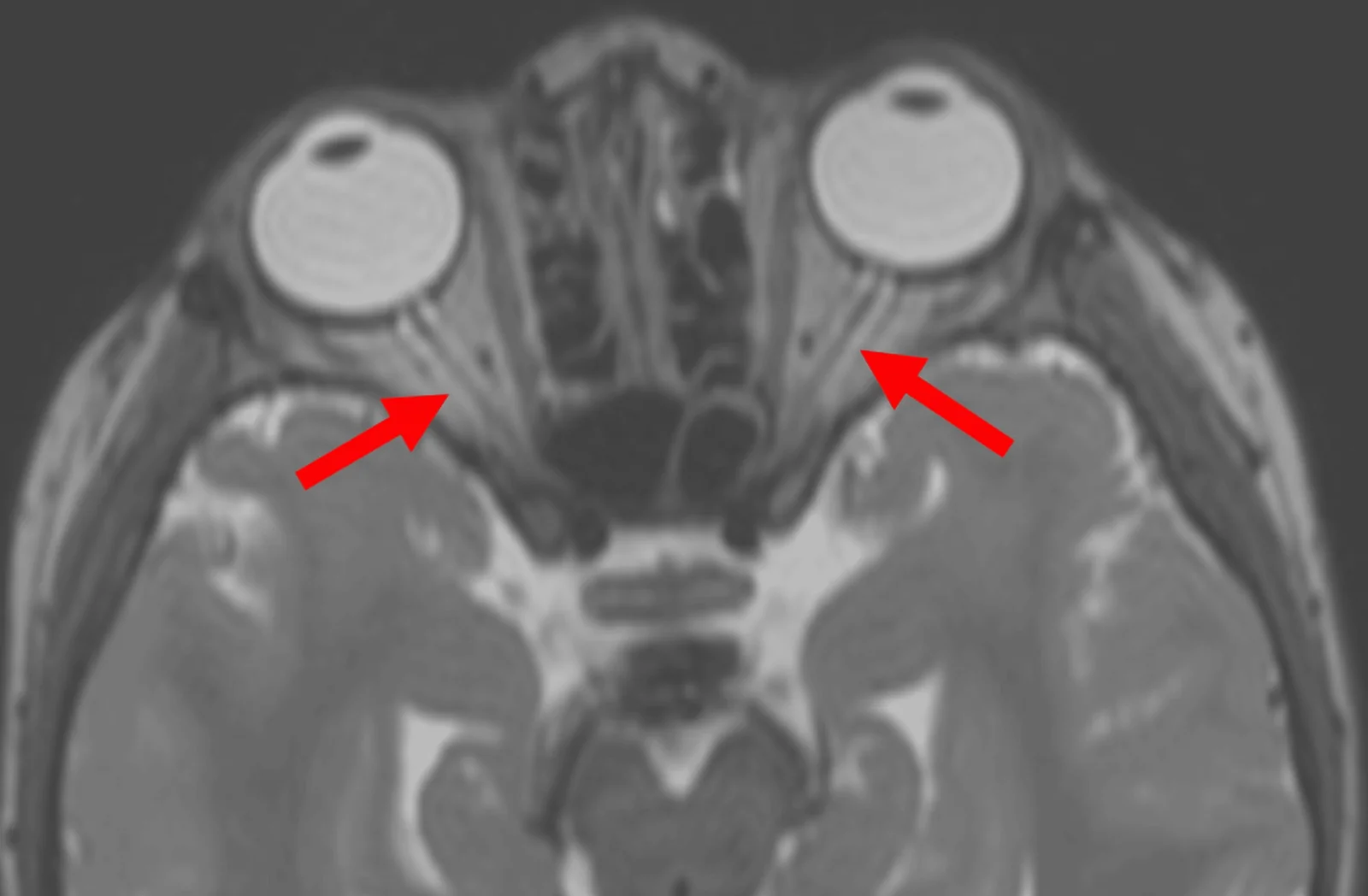
MRI image demonstrating bilateral optic nerve hypoplasia Axial short tau inversion recovery (STIR) MRI image of the patient shows bilateral hypoplastic optic nerves (red arrows).

**Figure 7:**
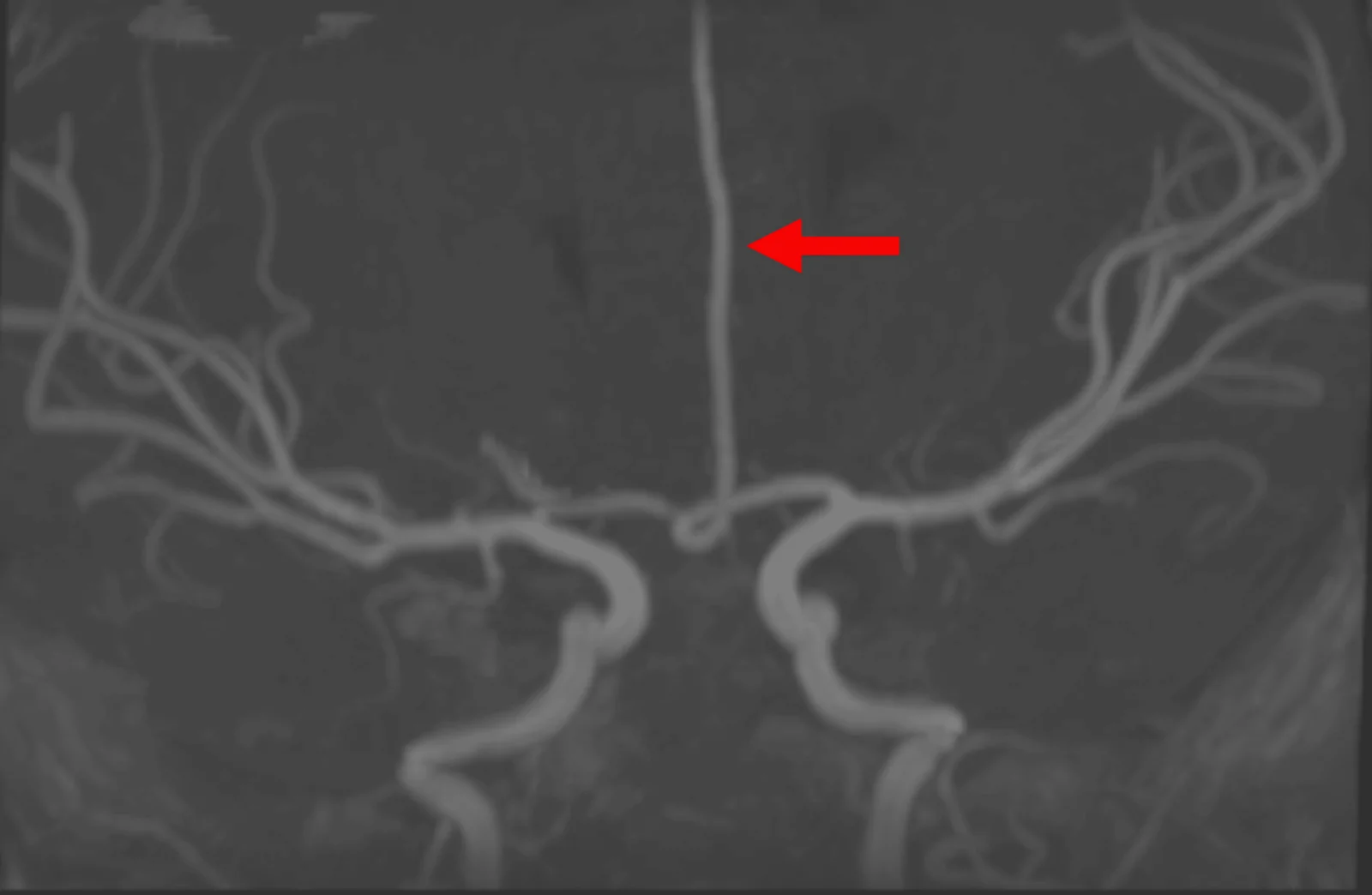
MRI image demonstrating an azygous anterior cerebral artery MR angiogram with 3D reconstruction shows an azygous anterior cerebral artery (red arrow).

**Figure 8:**
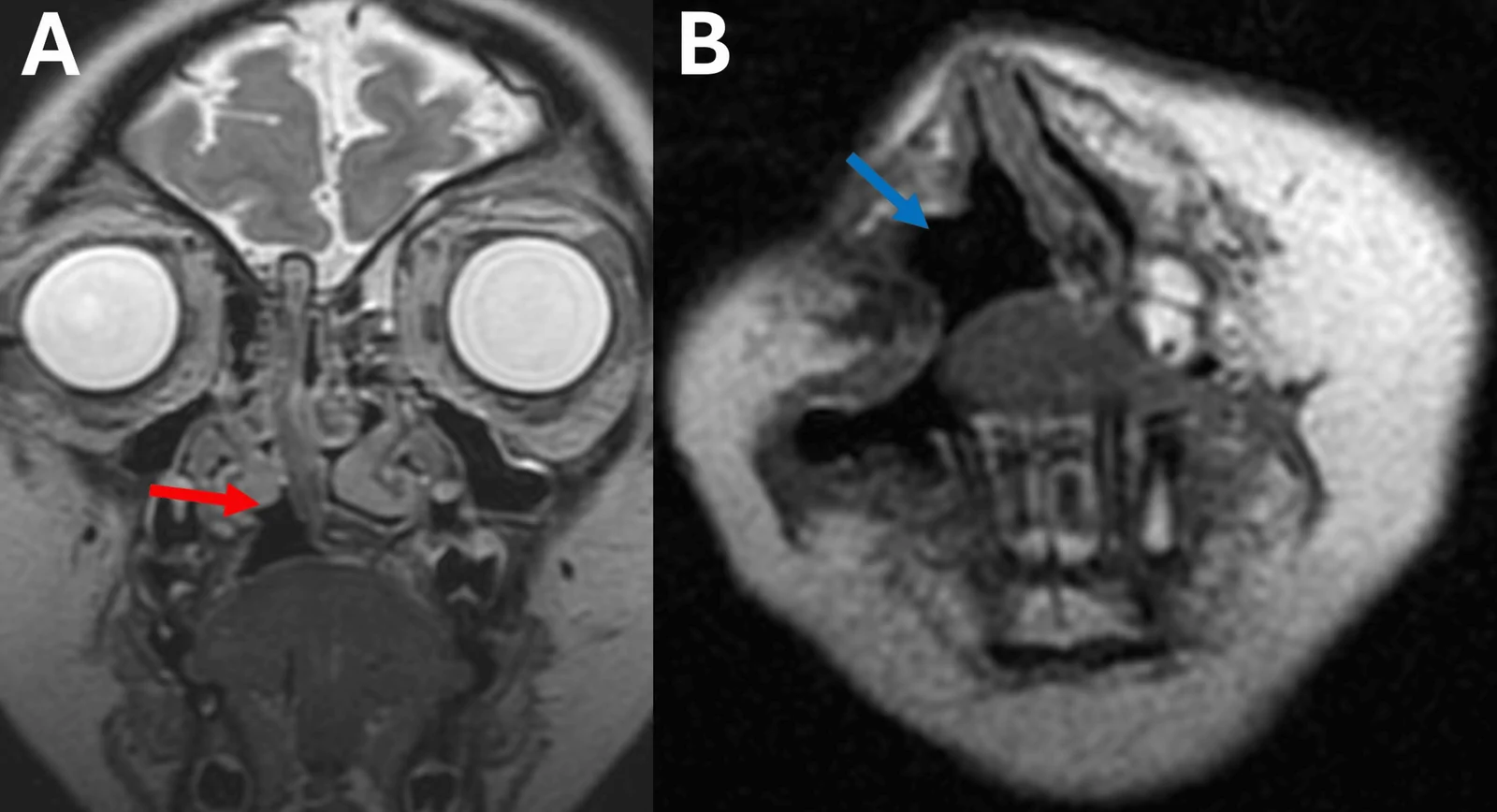
MRI images demonstrating cleft palate and cleft lip Coronal T2-weighted MRI image in the plane of the globes (A) shows cleft palate (red arrow). Coronal T2-weighted MRI image in the anterior plane (B) shows the cleft lip (blue arrow).

MRI of the brain without intravenous contrast demonstrated fusion of gyri across the midline involving the posterior parts of the frontal lobes (Figures 1, 2A) and anterior parts of the parietal lobes (Figures 3B, 4C), findings consistent with the MIH variant of HPE. The body of the corpus callosum was absent, and there was hypoplasia of the genu and splenium of the corpus callosum (Figure 5). Absence of the septum pellucidum (Figure 3) and optic nerve hypoplasia (Figure 6) were present, findings consistent with septo-optic dysplasia. The pituitary stalk and gland were normal. Bilateral migrational anomalies manifested by abnormal sulcations and polymicrogyria were noted (Figure 1). Additional findings included subependymal gray matter heterotopias along the bilateral frontal horns (Figures 4A, 4B), colpocephaly (Figure 2), and thinning of the periventricular white matter (Figure 2). An azygos anterior cerebral artery was present (Figure 7). Her cleft lip and palate were also evident on MRI (Figure 8). There was no fusion of the thalami or caudate nuclei.

## Discussion

Syntelencephaly is associated with a constellation of radiologic and clinical features that vary in prevalence and severity among reported cases, as will be discussed below. Our patient demonstrated many of the characteristic imaging features of syntelencephaly while also exhibiting bilateral optic nerve hypoplasia and pituitary dysfunction in the form of diabetes insipidus, together fulfilling the diagnostic criteria for septo-optic dysplasia. Many of the neuroimaging findings observed in this case are characteristic of syntelencephaly and have been described previously; however, their co-occurrence with features consistent with all three criteria for septo-optic dysplasia distinguishes this case from those reported in the literature. A literature search was conducted of PubMed and Google Scholar with combinations of "middle interhemispheric variant" or "syntelencephaly" and "septo-optic dysplasia," "pituitary gland dysfunction," "endocrine dysfunction," or "optic nerve hypoplasia." Although endocrine dysfunction has been reported in some patients with MIH, no published cases found in our literature search described the coexistence of MIH and optic nerve hypoplasia with endocrine dysfunction.

For example, Rajalakshmi et al. described a patient with separate basal ganglia and thalami, absence of the septum pellucidum, and agenesis of the body of the corpus callosum, findings that were also present in our patient. Unlike our patient, however, their patient had an intact genu and splenium and vertically oriented Sylvian fissures, whereas our patient exhibited hypoplasia of the genu and splenium with horizontally oriented Sylvian fissures. Optic nerve hypoplasia and pituitary dysfunction were not reported in their case (Table 1) [8]. Bulakbasi et al. reported a series of five patients with syntelencephaly, all of whom lacked a septum pellucidum. An azygos anterior cerebral artery was identified in three patients, and partial absence of the genu and splenium with complete absence of the body of the corpus callosum was observed in two patients, findings that were also present in our patient. In contrast, all five patients demonstrated a central ventricular notch extending into non-cleaved heterotopic gray matter involving the body of the corpus callosum, three had vertically oriented Sylvian fissures, and three had normal genu and splenium, features not observed in our patient. None of the patients had optic nerve abnormalities; however, one patient had an ectopic neurohypophysis and hypoplastic pituitary stalk (Table 1) [9]. A series by Simon et al. evaluating 21 patients with syntelencephaly identified several findings similar to those in our patient, including heterotopic gray matter or dysplastic cerebral cortex in 18 of 21 patients and an azygos anterior cerebral artery in all 16 patients in whom the vessel could be assessed. Among the 18 patients in whom the corpus callosum could be evaluated, some portion of the corpus callosum was present in all cases. The genu and splenium were both identified in 11 patients, four had either the genu or splenium (but not both), and three had a portion of the body in addition to the genu, splenium, or both. Sylvian fissures were abnormally connected across the midline over the vertex in 18 of 21 patients, and dorsal cysts were present in five patients, neither of which was observed in our patient. The optic chiasm appeared normal in 12 of the 13 patients in whom it could be assessed. Although one patient had a small, presumably hypoplastic optic chiasm, optic nerve hypoplasia was not reported in any patient in the series. The pituitary gland was visualized in 16 of 21 patients and was noted to be subjectively small in four patients (Table 1) [5].

**Table 1:** Comparison of clinical and neuroimaging findings between the present case and previously reported cases of syntelencephaly Articles referenced include Rajalakshmi et al. [8], Bulakbasi et al. [9], and Simon et al. [5].

Finding	Present Case	Rajalakshmi et al. (n = 1)	Bulakbasi et al (n = 5)	Simon et al. (n = 21)
Fusion of posterior frontal/anterior parietal lobes	Yes	Yes	Yes	Yes
Absent septum pellucidum	Yes	Yes	Yes	No
Hypoplastic/absent body of corpus callosum	Yes	Yes	Yes	No (five patients were unable to be assessed)
Hypoplastic/absent genu of corpus callosum	Yes	No	2/5	4/21 had either a splenium or genu but not both. Article does not specify further
Hypoplastic/absent splenium of corpus callosum	Yes	No	2/5	4/21 had either a splenium or genu but not both. Article does not specify further
Separate thalami	Yes	Yes	1/5 (two had abnormal orientation but were still separate)	7/21
Gray matter heterotopia or cortical dysplasia	Yes	No	5/5 had cortical dysplasia	18/21 had gray matter heterotopia, cortical dysplasia, or both
Polymicrogyria or migrational anomaly	Yes	No	No	No
Azygos anterior cerebral artery	Yes	No	3/5	16/21 (five patients were unable to be assessed)
Vertically oriented Sylvian fissures	No	Yes	3/5	18/21
Dorsal cyst	No	No	No	5/21 (one patient was unable to be assessed)
Optic nerve hypoplasia	Yes	No	No	No (one patient had a small optic chiasm that was presumed to be hypoplastic; optic nerve was otherwise not described)
Pituitary abnormality on MRI	No	No	1/5 had ectopic neurohypophysis and hypoplastic stalk	4/21 were subjectively small (five patients were unable to be assessed)
Endocrine dysfunction	Yes	No	Not reported	Not reported

Consistent with the rarity of pituitary dysfunction in the cases discussed above, other studies also suggest that endocrine abnormalities are uncommon in the middle interhemispheric variant (MIH) of holoprosencephaly despite being well-recognized features of the more classic forms of holoprosencephaly [1,10]. In a series of 15 patients with syntelencephaly, Lewis et al. reported no endocrinopathies [10]. In contrast, although our patient had a eutopic pituitary gland on MRI, she demonstrated pituitary dysfunction in the form of intermittent diabetes insipidus.

The coexistence of pituitary dysfunction and optic nerve hypoplasia in our patient raises the possibility of a shared developmental mechanism linking syntelencephaly and septo-optic dysplasia. Both disorders arise during an overlapping period of early forebrain morphogenesis, approximately between the fourth and eighth weeks of gestation, suggesting that their concurrence may reflect disruption of a common developmental process rather than two unrelated embryologic insults.

At approximately five weeks of gestation, the prosencephalon divides into the telencephalon and diencephalon [11]. Interhemispheric cleavage (approximately day 32) and falx differentiation (approximately day 56) occur in close temporal proximity to hypothalamic-pituitary induction, which is patterned during weeks 4 through 6 [12,13]. Optic nerve development follows a similar sequence, beginning with optic vesicle outpouching at approximately four weeks, followed by retinal ganglion cell differentiation at approximately six weeks and subsequent ingrowth of chiasmal fibers [14]. Failure of retinal ganglion cell development has been implicated in optic nerve hypoplasia [14]. Because these events occur during the same developmental window, a single disruptive event acting during early forebrain development could plausibly produce simultaneous but anatomically distinct midline abnormalities affecting the cerebral hemispheres, optic nerves, and hypothalamic-pituitary axis.

This embryologic overlap is further supported by shared developmental signaling pathways. Syntelencephaly is thought to result from localized disruption of dorsal roof plate and posterior midline patterning, with ZIC2 implicated in a subset of cases [12]. In a cohort of 509 patients with holoprosencephaly, Brown et al. identified 16 individuals with ZIC2 mutations, including one with the MIH variant [15]. The relatively mild in-frame deletion observed in that patient suggested that partial preservation of ZIC2 function may contribute to the less severe phenotype of syntelencephaly compared with classic forms of holoprosencephaly [15]. In contrast, septo-optic dysplasia has been associated with transcription factors involved in anterior midline and hypothalamic-pituitary development, including HESX1, SOX2, SOX3, OTX2, PROKR2, and FGF1/FGF8 [12,13]. Although these genes have distinct developmental roles, they function within interconnected dorsoventral and midline patterning networks that also involve SHH signaling [13,16,17]. Consequently, a genetic variant or environmental insult affecting these shared pathways could plausibly result in the combined phenotype observed in our patient.

Several authors have proposed that septo-optic dysplasia represents the mild end of the ventral induction and cleavage spectrum of holoprosencephaly, whereas others consider it a distinct disorder of midline development rather than a disorder of midline cleavage [11,12]. Regardless of classification, both models support the possibility that syntelencephaly and septo-optic dysplasia represent regional manifestations of a broader disturbance in early midline forebrain development.

Given the temporal overlap between interhemispheric cleavage, falx differentiation, and hypothalamic-pituitary-optic induction during the fourth through eighth gestational weeks, it is biologically plausible that a single disruption of midline developmental signaling produced both the interhemispheric fusion characteristic of syntelencephaly and the features of septo-optic dysplasia in our patient. However, because this report describes a single patient without confirmation of a shared molecular mechanism, causality cannot be established, and the coexistence of these conditions may alternatively represent two temporally coincident but mechanistically distinct developmental events.

Overall, our patient shares many of the hallmark neuroimaging features of syntelencephaly while also exhibiting bilateral optic nerve hypoplasia and pituitary dysfunction. To the best of our knowledge, this is the first reported case of syntelencephaly occurring in association with all three diagnostic features of septo-optic dysplasia, representing a novel finding in the published literature on syntelencephaly. This case highlights the importance of multidisciplinary care, including ophthalmologic and endocrinologic evaluation and longitudinal surveillance, in patients with syntelencephaly, particularly when clinical features raise concern for associated visual or pituitary abnormalities.

## Conclusions

We report a case of a five-year-old girl with syntelencephaly characterized by fusion of the cerebral hemispheres at the posterior parts of the frontal lobes and anterior parts of the parietal lobes, hypoplasia of the corpus callosum, heterotopic gray matter, an azygos anterior cerebral artery, and absence of the septum pellucidum. Our patient also met the diagnostic criteria for septo-optic dysplasia, with bilateral optic nerve hypoplasia and diabetes insipidus. We propose that this co-occurrence may reflect a shared disruption of midline forebrain development during a common window of early gestation, though causality cannot be established from a single case. Recognition of both the typical and atypical manifestations of syntelencephaly, including its potential association with septo-optic dysplasia, may facilitate earlier diagnosis, prompt evaluation for associated endocrine and ophthalmologic abnormalities, and encourage appropriate multidisciplinary management.
